# Supplementary material for: Tumor-infiltrating Leukocytes Suppress Local Inflammation Via Interleukin-1 Receptor Antagonist in a Syngeneic Prostate Cancer Model
Source: Biology (Basel). 2020 Mar 31;9(4):67. doi: 10.3390/biology9040067 (PMC7235745; doi:10.3390/biology9040067)
Supplement: Supplementary file 1 [file biology-09-00067-s001.pdf]

Tumor-infiltrating leukocytes suppress local inflammation via interleukin-1 receptor antagonist in a syngeneic prostate cancer model

Yu-Ching Fan, Wei-Yu Chen, Kuan-Der Lee, Yuan-Chin Tsai

Summary:
The supplementary information contains Supplementary Figure S1 and Supplementary Table S1

Supplementary Figure S1
Validation of Ficoll gradient, angiogenesis in tumor microenvironment, and analysis of clinical datasets

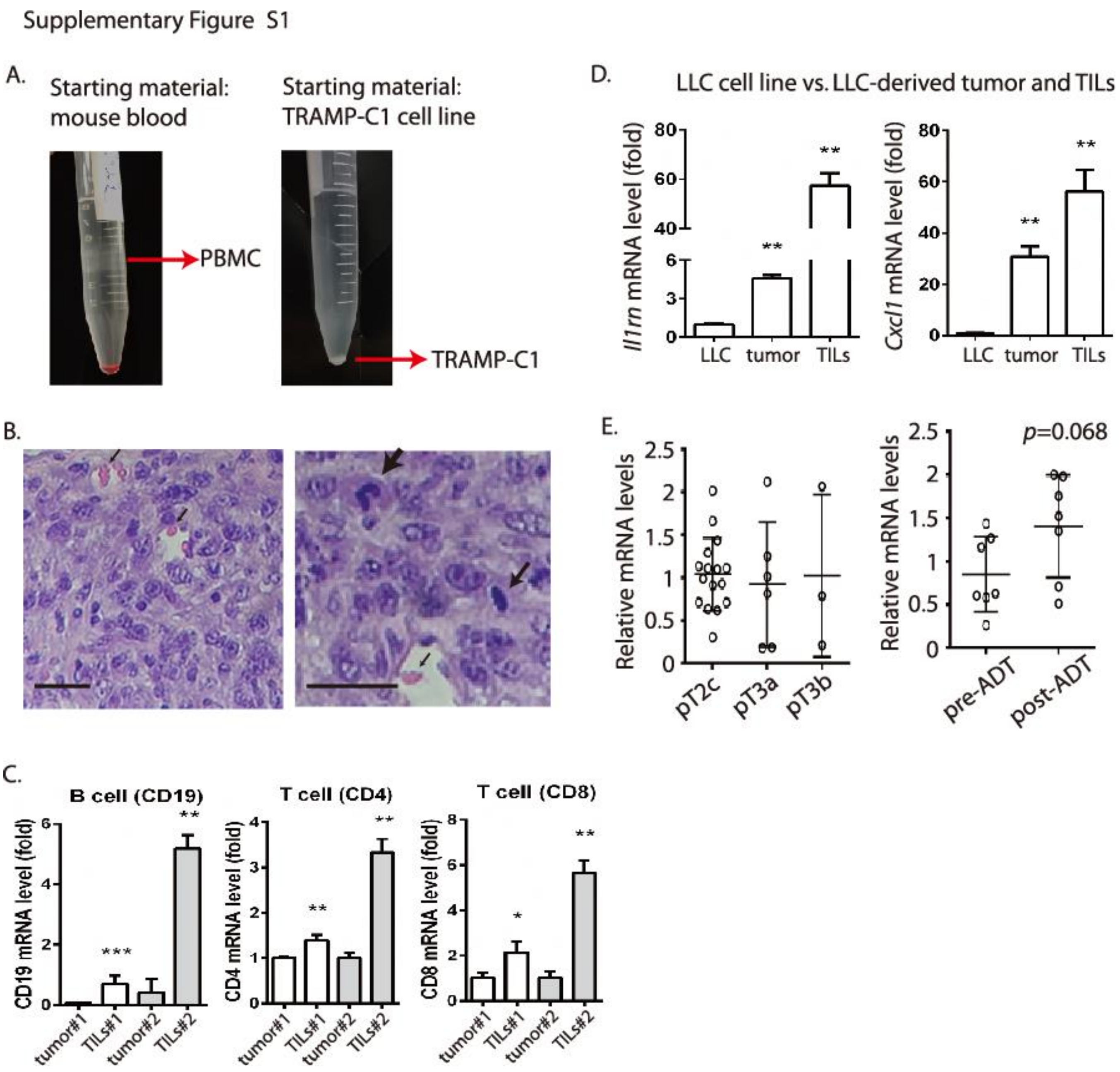

Figure legend

(A) Validation of Ficoll gradient procedure to separate leukocytes and cancer cells. Left panel: the peripheral blood mononuclear cells (PBMC) in mouse blood locate in the interphase (red arrow) after centrifugation. Right panel: TRAMP-C1 cells are pelleted down the bottom of the tube under the same centrifugation procedure.

(B) Selected images of tumor tissues (P3-T1, P3-T2) collected from immune competent mice after s.c. injection of P2-C primary cells. Big arrow: granulocytes with polymorphonuclear feature (left). Middle arrow: lymphocyte. Small arrows: capillary-like acini containing red blood cells. Scale bar: 10μm.

(C) Monitoring immune cell markers in tumor-derived samples by measuring B cell marker *Cd19* (left) and T cell markers *Cd4* (middle) and *Cd8* (right). qPCR measurements were derived from two replicates and results are presented as the mean ± SD. Student's t-test. * $p<0.05$, ** $p<0.01$, *** $p<0.001$.

(D) Lewis lung cancer (LLC)-derived tumor environment has elevated *Il1rn* and *Cxcl1* expression. mRNA levels were compared in the LLC cell line (LLC), its tumor tissue (tumor), and its derived TILs. qPCR measurements were derived from three technical replicates and results are presented as the mean ± SD. Student's t-test. * $p<0.05$, ** $p<0.01$, *** $p<0.001$, **** $p<0.0001$.

(E) Monitoring *IL1RN* mRNA levels in two clinical datasets. Left panel: 25 human prostate cancer samples with different tumor stages (pT2c: n=16, pT3a: n=6, pT3b: n=3) (Accession ID: SRP005908). Right panel: 14 human prostate cancer samples from 7 patients before (pre-ADT) and after (post-ADT) ADT treatment. (Accession ID: SRP026387).

Supplementary Table

Supplementary Table S1. Primer sequences for the qRT-PCR.

Gene	5'-3'
RELA F	CCG ACT TGT TTG GGT GAT CT
RELA R	TCC GTC TCC AGG AGG TTA AT
CCR2 F	TTA CAC CTG TGG CCC TTA TTT
CCR2 R	CTG AGT AGC AGA TGA CCA TGA C
CCR4 F	TCT TCC TGC CTC CTC TCT AC
CCR4 R	TGG ACT TGA GCC TCT TGT ATT T
IL1RN F	CAG CTC ATT GCT GGG TAC TT
IL1RN R	CTC AGA GCG GAT GAA GGT AAA G
CXCL1 F	GTG TCT AGT TGG TAG GGC ATA AT
CXCL1 R	CAG TCC TTT GAA CGT CTC TGT
CXCL10 F	AGT AAC TGC CGA AGC AAG AA

CXCL10 R	GCA CCT CCA CAT AGC TTA CA
CCL2 F	AGT AGG CTG GAG AGC TAC AA
CCL2 R	GTA TGT CTG GAC CCA TTC CTT C
GAPDH F	TCT CCC TCA CAA TTT CCA TCC
GAPDH R	GGG TGC AGC GAA CTT TAT TG
